# Coronary inflammation and cardiovascular risk in breast cancer after radiotherapy

**DOI:** 10.1093/eurheartj/ehaf260

**Published:** 2025-04-21

**Authors:** Christos P Kotanidis, Thomas Halborg, Pete Tomlins, Kenneth Chan, Sam Fry, Gaëlle Jimenez, Matthieu Lapeyre, Nikant Sabharwal, Keith M Channon, Stefan Neubauer, Sophie Jacob, Charalambos Antoniades

**Affiliations:** Acute Multidisciplinary Imaging and Interventional Centre, Division of Cardiovascular Medicine, Radcliffe Department of Medicine, University of Oxford, Level 6 West Wing, John Radcliffe Hospital, Headley Way, Headington, OX3 9DU Oxford, UK; Division of Cardiovascular Medicine, Heart and Vascular Center, Brigham and Women’s Hospital, Harvard Medical School, Boston, MA, USA; Acute Multidisciplinary Imaging and Interventional Centre, Division of Cardiovascular Medicine, Radcliffe Department of Medicine, University of Oxford, Level 6 West Wing, John Radcliffe Hospital, Headley Way, Headington, OX3 9DU Oxford, UK; Caristo Diagnostics Ltd, Oxford, UK; Acute Multidisciplinary Imaging and Interventional Centre, Division of Cardiovascular Medicine, Radcliffe Department of Medicine, University of Oxford, Level 6 West Wing, John Radcliffe Hospital, Headley Way, Headington, OX3 9DU Oxford, UK; Caristo Diagnostics Ltd, Oxford, UK; Department of Radiation Oncology, Clinique Pasteur, Toulouse 31076, France; Department of Radiology, Clinique Pasteur, Toulouse 31076, France; Department of Cardiology, NIHR Biomedical Research Centre, Oxford University Hospitals NHS Foundation Trust, John Radcliffe Hospital, Headley Way, Headington, OX3 9DU Oxford, UK; Acute Multidisciplinary Imaging and Interventional Centre, Division of Cardiovascular Medicine, Radcliffe Department of Medicine, University of Oxford, Level 6 West Wing, John Radcliffe Hospital, Headley Way, Headington, OX3 9DU Oxford, UK; Department of Cardiology, NIHR Biomedical Research Centre, Oxford University Hospitals NHS Foundation Trust, John Radcliffe Hospital, Headley Way, Headington, OX3 9DU Oxford, UK; Acute Multidisciplinary Imaging and Interventional Centre, Division of Cardiovascular Medicine, Radcliffe Department of Medicine, University of Oxford, Level 6 West Wing, John Radcliffe Hospital, Headley Way, Headington, OX3 9DU Oxford, UK; Department of Cardiology, NIHR Biomedical Research Centre, Oxford University Hospitals NHS Foundation Trust, John Radcliffe Hospital, Headley Way, Headington, OX3 9DU Oxford, UK; Laboratory of Epidemiology, Institute for Radiation Protection and Nuclear Safety (IRSN), Fontenay-Aux-Roses 92260, France; Acute Multidisciplinary Imaging and Interventional Centre, Division of Cardiovascular Medicine, Radcliffe Department of Medicine, University of Oxford, Level 6 West Wing, John Radcliffe Hospital, Headley Way, Headington, OX3 9DU Oxford, UK; Department of Cardiology, NIHR Biomedical Research Centre, Oxford University Hospitals NHS Foundation Trust, John Radcliffe Hospital, Headley Way, Headington, OX3 9DU Oxford, UK

**Keywords:** Radiotherapy, Cardio-oncology, Biomarkers, Coronary inflammation, Cardiovascular risk, Computed tomography imaging

## Introduction

Radiotherapy (RT) is known to reduce the rate of disease recurrence following breast-conserving surgery in patients with breast cancer, but also entails irradiation of the heart, which can lead to cardiotoxicity and vascular injury, specifically through sustained inflammation.^1^ During the last decades, RT advancements have been made in limiting the heart dose and the link between RT and long-term cardiovascular risk is less clear, with some older studies highlighting a dose-dependent increase in the subsequent rate of ischaemic heart disease,^2^ while more recent studies showed no evidence for increased cardiac mortality among women treated with RT.^3^ We sought to explore the evolution of coronary inflammation and residual inflammatory risk 2 years after RT in women with breast cancer.

## Methods

We included 105 breast cancer patients from the BACCARAT study treated with RT without chemotherapy and no prior history of other malignancy or cancer treatment.^4^ Coronary computed tomography angiography (CCTA) images were taken after surgery (mean interval of 46 ± 15 days) and before RT and 2 years following RT. No patient died or dropped out of the study due to adverse clinical events between follow-up visits. Four patients, who were initially screened and underwent the first CCTA imaging study, did not complete the 2-year follow-up scan for personal reasons. Calcium score and quantitative plaque characteristics were measured from the scans at baseline and at follow-up with CaRi-Research v2.5.6 using an unsupervised algorithm, and visually inspected in selected plaque cases to verify the results, while plasma inflammatory molecules were measured using ELISA for high-sensitivity C-reactive protein (hsCRP) and tumour necrosis factor alpha (TNFa). Coronary inflammation was measured in each coronary artery using the perivascular Fat Attenuation Index (FAI) Score, an established quantitative metric of coronary inflammation, interpreted in nomograms for age and sex.^5^ The patients’ 8-year risk for cardiac mortality was quantified using the AI-Risk prognostic model that integrates FAI Score with clinical risk factors (diabetes, smoking, hyperlipidaemia, and hypertension) and coronary plaque burden, as previously described^5^ and recently validated^6^ as part of a regulatory cleared medical device (CaRi-Heart^®^ v2.5). Indeed, this model provides accurate prediction of cardiac events at an individual patent level and captures well the inflammatory cardiovascular risk, as recently described in a large-scale external validation study.^6^

## Results

**Figure 1:**
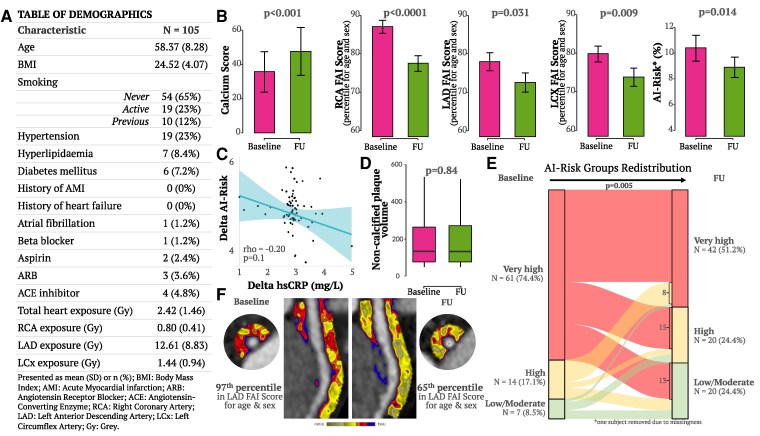
(*A*) Table of demographics for the study participants. (*B*) Calcium score was significantly increased 2 years after radiotherapy. Fat Attenuation Index Score (presented as percentiles on age and sex-specific nomograms) was significantly reduced 2 years after radiotherapy, suggesting reduction of coronary inflammation. The AI-Risk (8-year risk for cardiac mortality calculated by incorporating FAI Score, plaque burden and patient risk factors) was also significantly reduced 2 years after radiotherapy. (*C*) There was no correlation between delta-AI-Risk and delta serum high-sensitivity C-reactive protein from baseline to follow-up. (*D*) There was no change in the total non-calcified plaque volume between baseline (before radiotherapy) to follow-up (2 years after radiotherapy). (*E*) Alluvial plot of risk re-classification based on the AI-Risk classification system; (*F*) Example of the changes in perivascular fat attenuation index map around the proximal left anterior descending artery in a 50-year-old female patient with no history of cardiovascular disease. Values presented as mean ± SEM, and *P-*values derived by paired *t*-test or the Wilcoxon signed-rank test as appropriate; The values in the scatterplot have been transformed using the following formula: sqrt(variable − min(variable) + 1) and the *P-*value was derived by the Spearman’s rho test

The demographic characteristics and RT exposure information are presented in *Figure 1A*. At baseline, these women had significantly elevated inflammation in all the three coronary arteries, for their age [FAI Score in the right coronary artery (RCA) at the 87.05 ± 15.16 percentile, left anterior descending artery (LAD) at the 77.96 ± 21.03 percentile, and left circumflex artery (LCX) at the 79.82 ± 18.51 percentile of the reference population of the CaRi-Heart^®^ device]. Two years after RT, we found an increase in coronary calcification (*P* < .001 vs. baseline, *Figure 1B*), but, in spite of this, a parallel reduction of coronary inflammation, as captured by a significant reduction in FAI Score percentiles in the RCA (*P* < .0001 vs. baseline, *Figure 1B*), the left anterior descending (LAD, *P* = .031 vs. baseline, *Figure 1B*) and the left circumflex (LCX, *P* = .009 vs. baseline, *Figure 1B*). Two years after RT, the 8-year predicted risk for cardiac mortality (AI-Risk) was reduced from 10.41% at baseline to 8.92% (*P* = .014, *Figure 1B*). The radiation exposure of the heart was not correlated with the change of the AI-Risk from baseline to follow-up (rho = −0.01, *P* = .95). Similarly, there was no significant correlation between the radiation exposure of each coronary artery and the change of the FAI-Score percentile of the radiated vessel (rho = −0.03, *P* = .77 for RCA, rho = −0.21, *P* = .06 for LAD and rho = −0.14, *P* = .21 for LCX). These findings confirm that the reduction of coronary inflammation and the calculated inflammatory risk for cardiac mortality are not mediated by direct effects of the radiation on the coronary arteries or the heart, but are most likely mediated by the effects of the RT on the tumour itself, which could be the driver behind the observed changes. There were no significant changes in plasma levels of inflammatory molecules like hsCRP (from 1.90 ± 2.09 to 2.29 ± 2.82, *P* = .43) or TNFa (from 35.58 ± 12.17 to 34.62 ± 10.83, *P* = .53) from baseline to follow-up. No correlation was observed between delta-AI-Risk and either delta-hsCRP (rho = −0.20, *P* = .1, *Figure 1C*) or delta-TNFa (rho = −0.22, *P* = .06). No change was observed in total epicardial adipose tissue volume (*P* = .17) or non-calcified plaque volume (*P* = .84, *Figure 1D*). The AI-Risk at baseline was extremely high in this population, with nearly 75% of these women classified as having high 8-year risk for cardiac mortality at baseline. However, there was a striking risk re-classification 2 years after RT, with 45.9% of the high-risk patients reclassified to lower risk categories, and a parallel expansion of the ‘low’ risk population from 8.5 to 24.4% (*Figure 1E*). Additionally, to assess non-coronary inflammation, we utilized the radiotranscriptomic biomarker C19-RS, which captures cytokine-driven inflammation in the arterial wall of the thoracic descending aorta and the right internal mammary artery as previously validated.^7^ We observed a significant reduction in C19-RS in patients who had a reduction in their coronary inflammation levels at follow-up and had been redistributed to lower AI-risk categories (from 6.24 ± 1.43 to 5.46 ± 1.77, *P* = .037), as opposed to patients whose coronary inflammation levels remained similar or higher (from 6.06 ± 2.40 vs. 6.24 ± 2.05, *P* = .795).

## Discussion

Radiation-induced vascular injury is believed to promote atherosclerotic disease, particularly at high doses^8^ and in experimental models.^9^ Atherogenesis is driven by inflammation, and anti-inflammatory agents like canakinumab (an anti-IL-1b monoclonal antibody) or colchicine prevent cardiovascular events. Although high-dose radiation affects the initiation, progression, and stability of atherosclerotic plaques, low and moderate doses may lead to a reduced inflammatory response of plaques and lesion size.^10^ Recently, 5 Gy cardiac radiation was shown to improve cardiac remodelling in a murine heart failure model and in patients with heart failure and ventricular tachycardia treated with cardiac RT.^11^ It is therefore important to understand the relationships between RT, coronary inflammation, and cardiovascular risk in women treated for breast cancer.

This study confirmed that women with active breast cancer experience elevated risk for cardiac mortality, driven by high inflammation observed in all three coronary arteries before they receive any treatment. Two years after RT, baseline coronary inflammation (measured by FAI Score in any of the three coronary arteries^6^) and resulting inflammatory risk (estimated using the AI-Risk model^6^) were decreased, independent from the local cardiac radiation exposure (*Figure 1F*). There was a significant shift of the distribution of FAI Score in these women to high percentiles at baseline [median(interquartile range (IQR)) 84(75–94) for LAD, 84(75–94) for LCX and 92(83–97) for RCA, which was reduced by Year 2 (80(58–91) for LAD, 78(58–91) for LCx, and 81(71–92) for RCA], although remained significantly higher compared with a reference cohort of women matched for age and cardiovascular risk factors hypertension, hypercholesterolaemia, smoking, and diabetes from the ORFAN registry^6^ [40(19–64) for LAD, 47(23–72) for LCX and 42(15–67) for RCA, *P* < .001 for all comparisons vs. baseline or 2 years post-RT]. The parallel increase of coronary calcification suggests plaque stabilization as coronary inflammation is resolved and supports the notion that high coronary inflammation observed at baseline may be driven by the tumour itself (explaining the high cardiovascular risk in these women at baseline), and it is reduced as the tumour is treated with RT. This is further supported by the parallel reduction of C19-RS, a radiotranscriptomic biomarker of cytokine-driven inflammation in the aorta and the right internal mammary artery. The interplay between cancer and inflammation has long been suggested to play a pivotal role in the biological processes that influence all stages of cancer development. Indeed, reports of reduction in circulating IL-4, IL-6, and IL-10 levels post-cancer treatment with chemotherapy in breast cancer patients^12^ proposed that tumour regression has the potential to attenuate the activity of the immune system, which could explain the reduction in coronary inflammation observed here after cancer treatment with RT. Surgery has been shown to induce an inflammatory response in the post-operative period, however its effect is primarily related to localized immune responses rather than direct effects on coronary arteries, and any systemic inflammatory response seems to settle within a month after surgery.^13^ Although this study did not perform blood sampling before surgery, the plasma levels of inflammatory biomarkers before RT (performed at least 1 month after surgery) were not higher than the levels of these plasma biomarkers at follow-up (2 years), confirming that any surgery-related systemic inflammatory response had settled by the time of the baseline CCTA. Further using the inherent nomograms from the CaRi-Heart^®^ v2.5 device is that we consider the reference cohort (CRISP-CT) representative of the ‘general population’, which may lead to underestimation of the actual inflammation in the BACCARAT cohort. Finally, this study focused on changes in coronary inflammation (delta-FAI) and cardiovascular risk (delta-AI-risk) without incorporating cancer prognosis or mortality, as our primary aim was to study inflammation in the context of cancer treatment.

## Conclusions

In conclusion, this report highlights a significant reduction in coronary inflammation and residual inflammatory risk, as captured in CCTA imaging in women with breast cancer 2 years after RT. However, our study is limited by a small sample size, emphasizing the need for larger cohort studies with longer follow-up, to better understand the true effects of RT on cardiovascular risk in breast cancer patients.

## Declarations

### Data Availability

Individual participant-level data used for this report are not publicly available, because they contain protected patient health information. Requests for data access should be directed to the corresponding author via email.
